# The early life course-related traits with three psychiatric disorders: A two-sample Mendelian randomization study

**DOI:** 10.3389/fpsyt.2023.1098664

**Published:** 2023-03-21

**Authors:** Renke He, Jiaying Mo, Kejing Zhu, Qinyu Luo, Xueying Liu, Hefeng Huang, Jianzhong Sheng

**Affiliations:** ^1^International Institutes of Medicine, The Fourth Affiliated Hospital, Zhejiang University School of Medicine, Yiwu, China; ^2^Department of Reproductive Endocrinology, Women’s Hospital, School of Medicine, Zhejiang University, Hangzhou, China; ^3^Key Laboratory of Reproductive Genetics, Ministry of Education, School of Medicine, Zhejiang University, Hangzhou, China; ^4^Shanghai Frontiers Science Center of Reproduction and Development, Shanghai, China; ^5^Research Units of Embryo Original Diseases, Chinese Academy of Medical Sciences, Shanghai, China; ^6^Shanghai Key Laboratory of Embryo Original Diseases, Shanghai, China

**Keywords:** birth weight, early life body size, body mass index, depression, attention deficit hyperactivity disorder, Alzheimer’s disease, Mendelian randomization

## Abstract

**Objectives:**

Several studies have indicated a potential association between early life course-related traits and neurological and psychiatric disorders in adulthood, but the causal link remains unclear.

**Methods:**

Instrumental variables (IVs) that have been shown to be strongly associated with exposure were obtained from summary data of genome-wide association studies (GWASs). Four early life course-related traits [i.e., birthweight (BW), childhood body mass index (BMI), early body size, and age at first birth (AFB)] were used as exposure IVs to estimate their causal associations with three neurological and psychiatric diseases [i.e., Alzheimer’s disease (AD), major depressive disorder (MDD), and attention-deficit hyperactivity disorder (ADHD)]. Four different statistical methods, i.e., inverse-variance weighting (IVW), MR–Egger (MRE), weighted median (WM), and weighted mode (Wm), were performed in our MR analysis. Sensitivity analysis was performed by using the leave-one-out method, and horizontal pleiotropy was assessed using the MR-PRESSO package.

**Results:**

There was evidence suggesting that BW has a causal effect on AD (OR_MR-PRESSO_ = 1.05, *p* = 1.14E-03), but this association was not confirmed *via* multivariable Mendelian randomization (MVMR) (OR_MVMR_ = 0.97, 95% CI 0.92–1.02, *p* = 3.00E-01). A strong relationship was observed between childhood BMI and ADHD among both sexes; a 1-SD increase in BMI significantly predicted a 1.46-fold increase in the OR for ADHD (*p* = 9.13E-06). In addition, a similar relationship was found between early life body size and ADHD (OR_MR-PRESSO_ = 1.47, *p* = 9.62E-05), and this effect was mainly driven by male participants (OR_MR-PRESSO_ = 1.50, *p* = 1.28E-3). Earlier AFB could significantly predict a higher risk of MDD (OR_MR-PRESSO_ = 1.19, *p* = 1.96E-10) and ADHD (OR_MR-PRESSO_ = 1.45, *p* = 1.47E-15). No significant causal associations were observed between the remaining exposures and outcomes.

**Conclusion:**

Our results reveal the adverse effects of childhood obesity and preterm birth on the risk of ADHD later in life. The results of MVMR also show that lower BW may have no direct relationship with AD after adjusting for BMI. Furthermore, AFB may predict a higher risk of MDD.

## Introduction

The “developmental origins of adult health and disease hypothesis” (DOHAD) was first proposed in the 20th century to explain how adverse effects during early development can cause permanent changes in physiology and metabolism, ultimately leading to an increased risk of diseases in adulthood (1). This theory was first applied and widely studied in metabolic and cardiovascular diseases (2, 3). As the theory progressed, it posited that early life traits are associated with a higher risk of adult mental diseases (4–6), including schizophrenia (SCZ), major depressive disorder (MDD), cognitive impairment, and attention-deficit hyperactivity disorder (ADHD).

Numerous studies have shown a significant relationship between early life-related traits and neurological and psychiatric diseases in adulthood, which may be due to increased oxidative stress in the central nervous system (CNS) (7) during early life. For instance, lower birth weight (BW) is associated with ADHD (8), anxiety, depression (9, 10), social problems (11–14), and Alzheimer’s disease (AD) (15, 16). Preterm birth (PTB) is associated with neurocognitive impairment (17). In addition, the increasing prevalence of adiposity among children is a serious public health concern, and the complex association between childhood obesity and mental health in adulthood is suggested to strengthen the severity and interdependency of each factor (18, 19). Mental diseases among adults have been proven to be associated with overweight and obesity in childhood (20), such as MDD (21) and ADHD (22, 23). In the past 30 years, the global number of mental disorders has increased from 80.8 million to 1.25 billion (24); in particular, mental disorders have had a considerable influence on the burden of the economy on both families and society (25, 26). Therefore, demonstrating the causal effects of early life traits on mental illness and estimating the magnitude of such effects would provide evidence to guide the development of health policies and would advance the therapeutic window to the prenatal period, thus helping to overcome and mitigate the human, social, and economic costs of mental illness (27). However, the relationship between early life traits on mental illness in adulthood remained unclear, and previous results have been criticized due to bias, pleiotropy, and common confounders of observational studies, which may reflect false information (28–30).

Two-sample Mendelian randomization (TSMR)—a novel analysis method for estimating the causal link between exposures and outcomes based on genome-wide association study (GWAS) datasets—offers an unprecedented challenge to classical epidemiology by revealing a causal relationship at the genetic level rather than the population level. MR analysis includes three basic assumptions. First, instrumental variables (IVs) should have a strong association with the exposures of interest. Second, selected IVs should remain independent of confounders related to exposures and outcomes. Third, genetic IVs should only affect the outcome through exposure rather than other alternative ways (31). Randomized controlled trials (RCTs) are usually considered the gold standard for proving a scientific hypothesis in clinical research. The basic principle of TSMR is similar to that of RCT, as the alleles of genetic variants are assorted randomly and independently (based on Mendel’s law) by eggs and sperm during the pregnancy period, which makes genes independent from confounders such as environmental or racial factors. In short, the results of TSMR are considered to be reliable.

Recently, MR studies have provided evidence of a positive association between BW and ADHD (32, 33), while an inverse correlation was confirmed between life course adiposity and AD (34), ADHD (35), and MDD (36). Herein, lower birth weight (LBW), childhood BMI, early life body size, and age at first birth (AFB) were described as early life course-related metrics, and we assessed the causal link between these four variables and three common neurological and psychiatric diseases in later life. The results remained stable, significant, and robust even after considering the influence of heterogeneity, sensitivity, and horizontal pleiotropy.

## Materials and methods

### Selection of outcome GWAS (AD, ADHD, and MDD)

To obtain comprehensive and reliable results regarding the causal link between early life traits and the incidence of mental disorders in adulthood, the largest and most commonly cited GWAS were selected. A meta-analyzed GWAS of AD among individuals with European ancestry was included in our study (total *n* = 455,258, cases *n* = 71,880, and controls *n* = 383,378). The first group consisted of 24,087 patients who were clinically diagnosed with late-onset AD and paired with 55,058 controls. The second phase collected 47,793 AD-by-proxy phenotype cases and 328,320 controls based on the UK Biobank (UKB) for whom parental AD status was available (37, 38). The summarized statistics for MDD were collected from a genome-wide meta-analysis consisting of seven cohorts, including 480,359 participants (cases *n* = 135,458 and controls *n* = 344,901) (39). In addition, a meta-analyzed dataset of ADHD was obtained from 12 cohort studies, which included 20,183 cases and 35,191 controls (40). Furthermore, we collected sex-specific ADHD GWAS data from the Psychiatric Genomics Consortium and iPSYCH Project and Swedish population register data (male-only GWAS: cases *n* = 14,154 and controls *n* = 17,948; female-only GWAS: cases *n* = 4,945 and controls *n* = 16,246) (41) (see Table 1 and Supplementary Tables S1–S8 for details).

**Table 1:** Overview of exposure and seven outcomes GWAS.

Traits	Consortium	(Case/Control) Sample Size	PMID
**Exposure**			
Birth weight (BW)	Early Growth Genetics (EGG)	153,781	27,680,694
Childhood BMI	Genome-Wide Meta-Analysis	61,111	33,045,005
Early life body size	UK Biobank, GWAS consortiums	453,169	32,376,654
Age at first birth (AFB)	UK Biobank	542,901	34,211,149
**Outcomes**			
Alzheimer’s disease (AD)	Genome-Wide Meta-Analysis	(71,880/383,378)455,258	30,617,256
Major depressive disorder (MDD)	Genome-Wide Meta-Analysis	(135,458/344,901)480,359	29,700,475
Attention deficit and hyperactivity disorder (ADHD)	Genome-Wide Meta-Analysis	(20,183/35,191)55,374	30,478,444
ADHD-female	Psychiatric Genomics Consortium and iPSYCH Project and Swedish population register data	(4,945/16,246)21,191	29,325,848
ADHD-male		(14,154/17,948)32,102	29,325,848
Early life body size	UK Biobank	454,718	ukb-b-4,650

### Selection of exposure IVs (BW, childhood BMI, early life body size, and AFB)

Based on research from the Early Growth Genetics (EGG) Consortium (42), 47 independent single-nucleotide polymorphisms (SNPs) were filtered from the largest GWAS as IVs representing BW to conduct TSMR analysis, and a significant association was observed (*p* < 5.0E-08). To date, the EGG consortium study has collected data from more than 30 studies, including 153,781 cases and 16,245,523 imputed SNPs. The sources of data include results of measurement at birth by medical practitioners, obstetrics records, medical and interview registers, and self-reports with mothers and adults in each study. Sex-specific BW was transformed into a z-score, and qualification control was based on the following exclusion criteria: extreme BW value, i.e., more than 5 SD of the average mean value; monozygotic or polyzygotic siblings; and preterm births, i.e., gestational age less than 37 weeks at birth. Exposure to SNPs of childhood BMI was identified from a GWAS meta-analysis containing 41 studies with a total sample size of 61,111 European children aged 2–10 years (43). Based on the UKB prospective cohort study and four GWAS consortiums, early life body size-related SNPs were gained from 453,169 participants (44, 45). All data were collected from clinical examinations, detailed information on self-reported health characteristics, genome-wide genotyping, and testing of biological samples (46). BMI was obtained using height (measured and recorded in meters) and weight (kilogram) measured at baseline and calculated as follows: kg/m^2^. Participants also answered the following question: “When you were 10 years old, would you have defined your body shape as thinner, plumper, or about average?” This item was used to assess early life body size. Exposure to AFB was determined from the largest meta-analysis of GWAS, including 542,901 participants from 36 cohort studies of European ancestry (47). All selected exposure SNPs are shown in Table 1, and detailed information on the meta-analysis is displayed in Supplementary Tables S1–S8.

Then, instrument variables were removed from the linkage disequilibrium (LD) after conducting the process of clumping (*R^2^* < 0.001, window size = 10,000 kb) to ensure their independence. Third, the robustness of the results was based on the sufficient power of IVs that were applied in our TSMR analysis, and all IV-SNPs were confirmed to have a strong effect (overall F-statistics value >10) by performing the F-statistics. The SNP of IVs for lower BW or earlier AFB was presented by supplying a negative sign on the estimated BW/AFB effect (42). Finally, these stringently collected SNPs were used as the IVs for subsequent TSMR analysis.

### Statistical analyses

We conducted a TSMR analysis in R software (version 4.0.3 for Mac) using the two-sample MR and MR-PRESSO packages (48, 49). We used different MR tools with distinct strengths and assumptions. The inverse-variance weighted (IVW) approach was chosen as the main tool for estimating causality (50). Estimates of correlations of early life-related traits with AD, ADHD, and depression from large GWAS were obtained by applying the IVW method with the fixed effects model. The weighted median (WM) method, MR–Egger (MRE) regression, weighted mode, and Mendelian Randomization Pleiotropy RESidual Sum and Outlier (MR-PRESSO) were also conducted as supplementary analyses to ensure the stability and reliability of our results (51). Assuming over 50% weights from valid IVs, the WM method provides consistent estimates of associations (52).

MR–Egger regression allows the existence of unbalanced pleiotropy, which relaxes the assumptions of MR and causes low power (53). However, the validity of the results in our TSMR analysis was demonstrated when these methods maintained consistency in both direction and effect size. MR-PRESSO analysis was used to detect possible outliers and eliminate bias due to horizontal pleiotropy, and correct results were obtained from MR-PRESSO analysis after removing the outlier SNPs (54). In other words, comparing the distance of a single SNP to the fitted line, the larger the distance, the more likely the SNP is to be an outlier and the more likely it is to exhibit horizontal pleiotropy; such SNPs need to be excluded (51). The level of heterogeneity was quantified by Cochran’s Q-test and I^2^ statistics, and significant heterogeneity was identified by a *p*-value of <0.05. In addition, sensitivity analysis was performed using the leave-one-out method to identify whether the effects of causal relationships would disappear when any single SNP was excluded. Furthermore, a multivariable MR procedure was performed after adjusting for the main determinants of exposure and outcome to reduce bias. All results are displayed in forest plots, scatterplots, leave-one-out plots, and funnel plots. Causal associations with *p*-values <4.16E-03 (*P*_Bonferroni correction_ = 0.05/12) were deemed statistically significant after Bonferroni correction for four exposures and three outcomes (55). *p*-values between 4.16E-03 and 5E-02 were considered to suggest evidence of causal associations. Because a positive genetic relationship of BW with BMI was considered, the causal association of LBW with AD was estimated by applying the multivariable MR analysis (MVMR) model. All results are presented as odds ratios (ORs) with 95% confidence intervals (CIs) (Supplementary Tables S18–S20).

## Results

### Two-sample Mendelian randomization analysis for the causal association between lower BW and three neurological and psychiatric diseases

**Figure 1:**
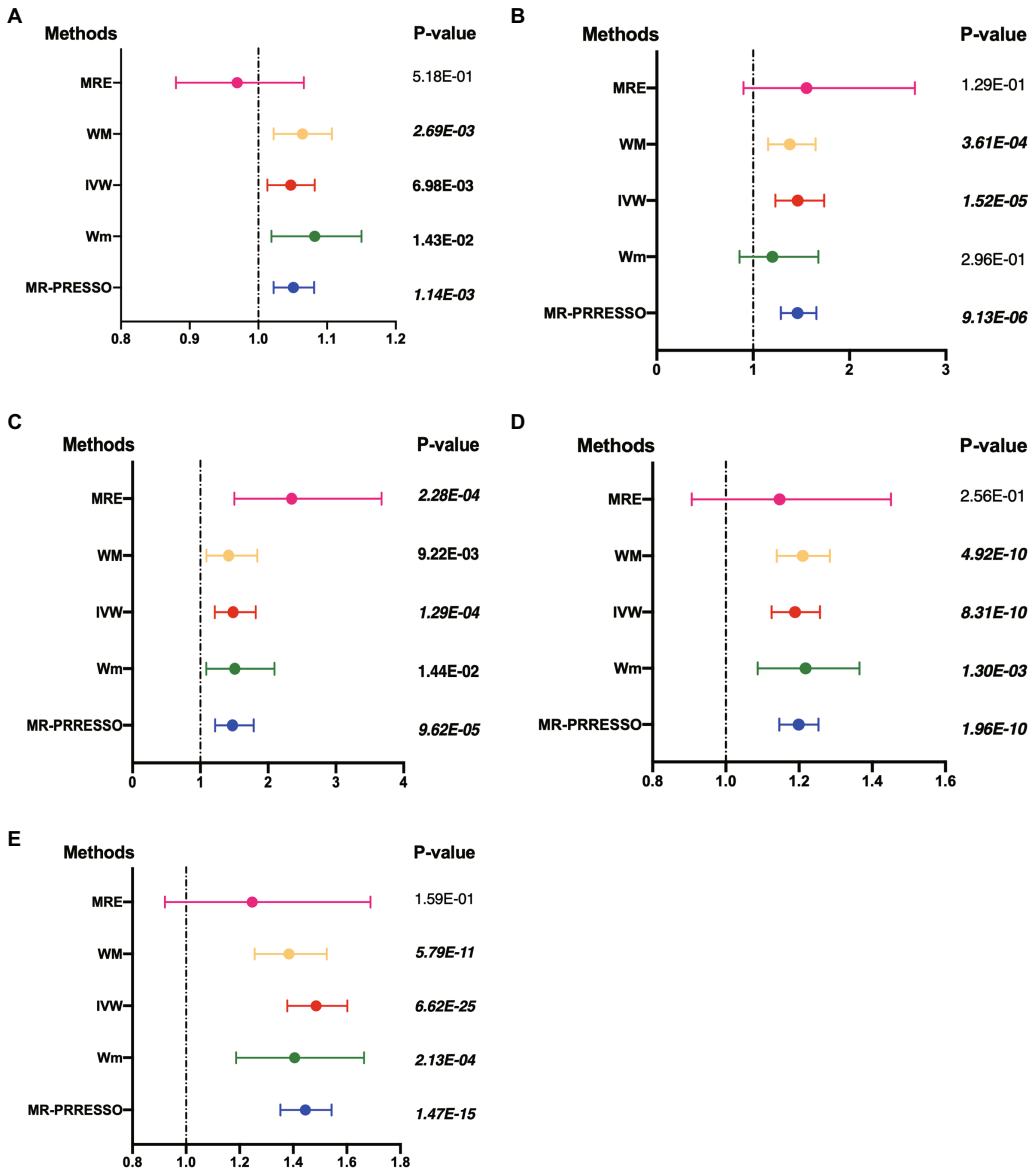
Results of four different methods of MR analysis. The MR analysis shows the effect of the exposure SNPs on the outcomes. **(A)** Lower birth weight (BW)-Alzheimer’s disease (AD); **(B)** childhood body mass index (BMI)-attention-deficit hyperactivity disorder (ADHD); **(C)** early life body size-ADHD; **(D)** age at first birth (AFB)-major depressive disorder (MDD); **(E)** AFB-ADHD; the solid dot means the causal effects of exposure on outcomes with four methods [MR-Egger (MRE); weighted median (WM); weighted mode (Wm); inverse-variance weighting (IVW); MR-Egger and Mendelian Randomization Pleiotropy RESidual Sum and Outlier (MR-PRESSO)]. The results of the binary outcomes are shown by OR [95%CI]. Numbers in bold mean *P-*values <5.00E-02, and italic and bold font means *P-*values <4.16E-03.

**Figure 2:**
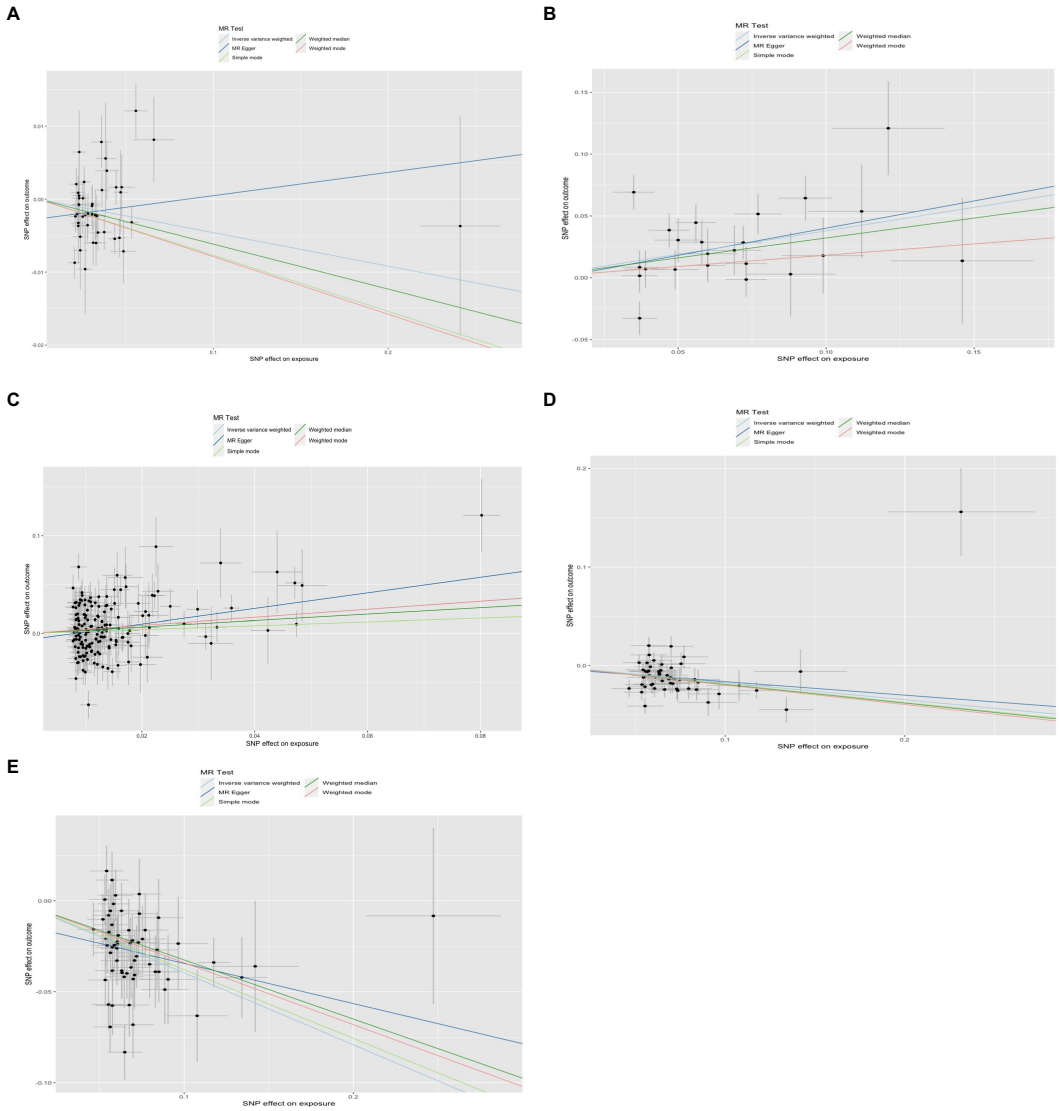
Results of the scatterplot. **(A)** Lower birth weight (BW)-Alzheimer’s disease (AD); **(B)** childhood body mass index (BMI)-attention-deficit hyperactivity disorder (ADHD); **(C)** early life body size-ADHD; **(D)** age at first birth (AFB)-major depressive disorder (MDD); **(E)** AFB-ADHD; the solid dot means the causal effects of exposure on outcomes with four methods [MR-Egger (MRE); weighted median (WM); weighted mode (Wm); inverse-variance weighting (IVW); MR-Egger and Mendelian Randomization Pleiotropy RESidual Sum and Outlier (MR-PRESSO)]. The results of the binary outcomes are shown by OR [95%CI]. Numbers in bold mean *P-*values <5.00E-02, and italic and bold font means *P-*values <4.16E-03.

**Figure 3:**
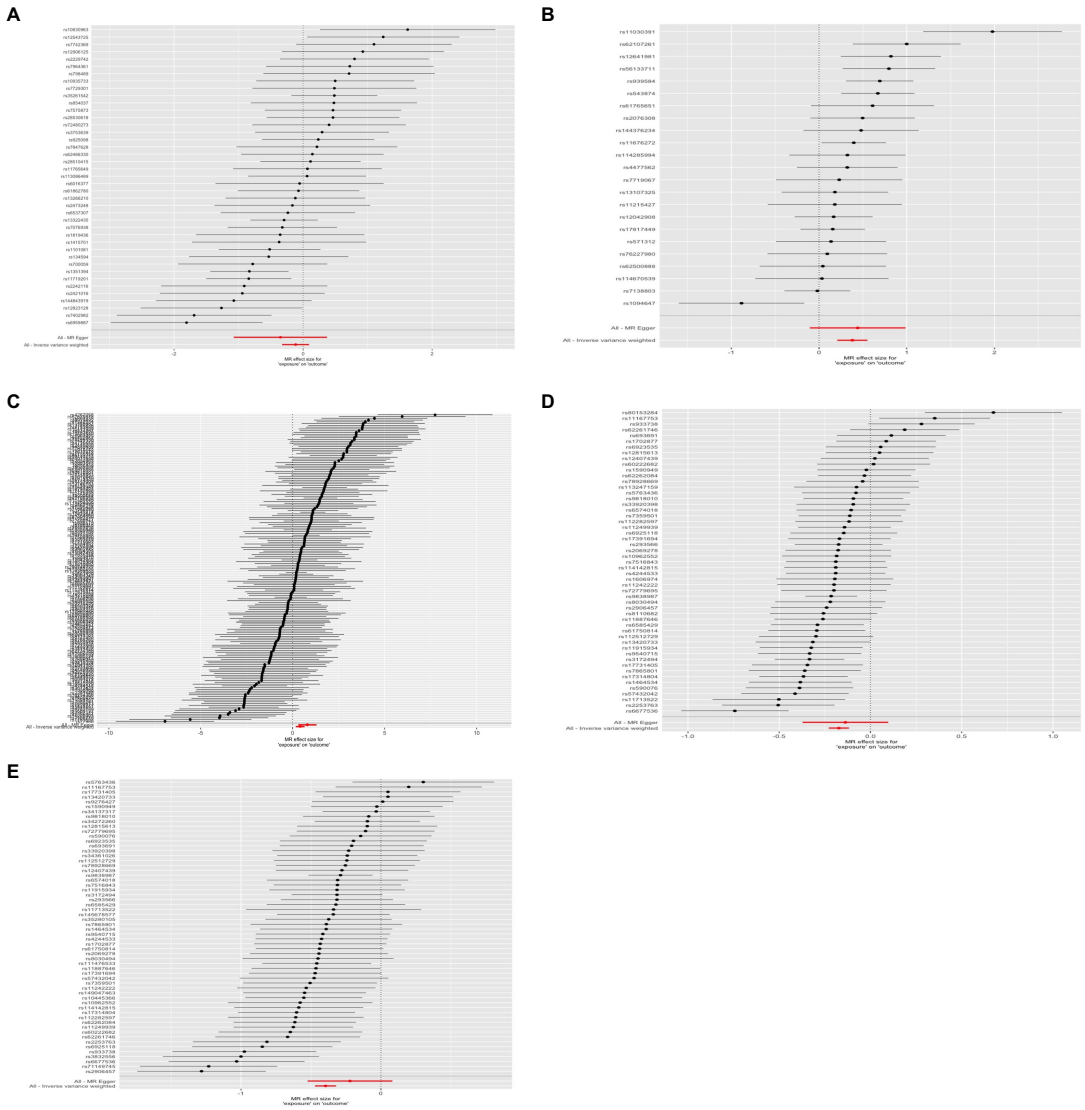
Results of the forest plot. **(A)** Lower birth weight (BW)-Alzheimer’s disease (AD); **(B)** childhood body mass index (BMI)-attention-deficit hyperactivity disorder (ADHD); **(C)** early life body size-ADHD; **(D)** age at first birth (AFB)-major depressive disorder (MDD); **(E)** AFB-ADHD; the solid dot means the causal effects of exposure on outcomes with four methods [MR-Egger (MRE); weighted median (WM); weighted mode (Wm); inverse-variance weighting (IVW); MR-Egger and Mendelian Randomization Pleiotropy RESidual Sum and Outlier (MR-PRESSO)]. The results of the binary outcomes are shown by OR [95%CI]. Numbers in bold mean *P-*values <5.00E-02, and italic and bold font means *P-*values <4.16E-03.

**Figure 4:**
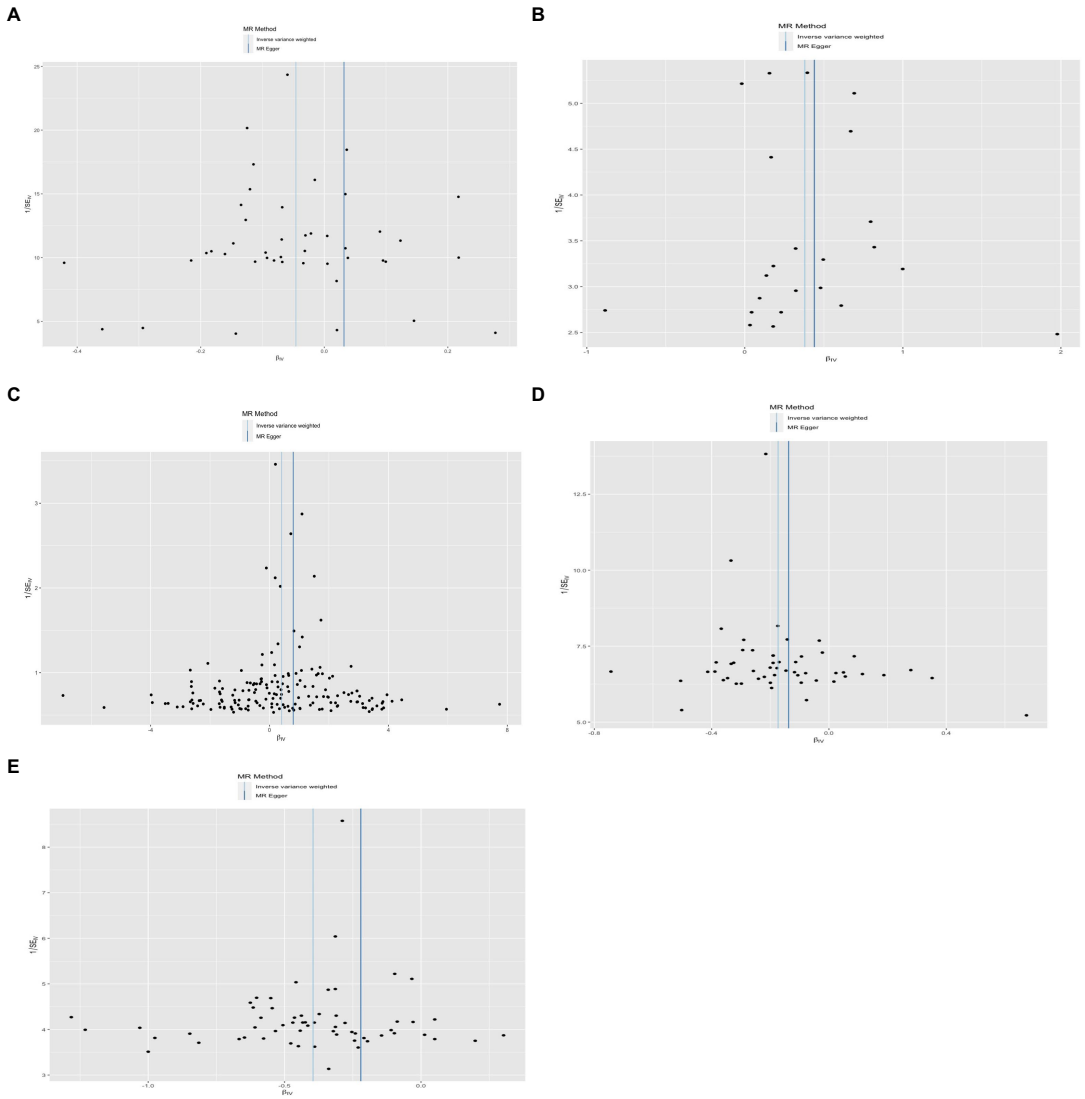
Results of the funnel plot. **(A)** Lower birth weight (BW)-Alzheimer’s disease (AD); **(B)** childhood body mass index (BMI)-attention-deficit hyperactivity disorder (ADHD); **(C)** early life body size-ADHD; **(D)** age at first birth (AFB)-major depressive disorder (MDD); **(E)** AFB-ADHD; the solid dot means the causal effects of exposure on outcomes with four methods [MR-Egger (MRE); weighted median (WM); weighted mode (Wm); inverse-variance weighting (IVW); MR-Egger and Mendelian Randomization Pleiotropy RESidual Sum and Outlier (MR-PRESSO)]. The results of the binary outcomes are shown by OR [95%CI]. Numbers in bold mean *P-*values <5.00E-02, and italic and bold font means *P-*values <4.16E-03.

**Figure 5:**
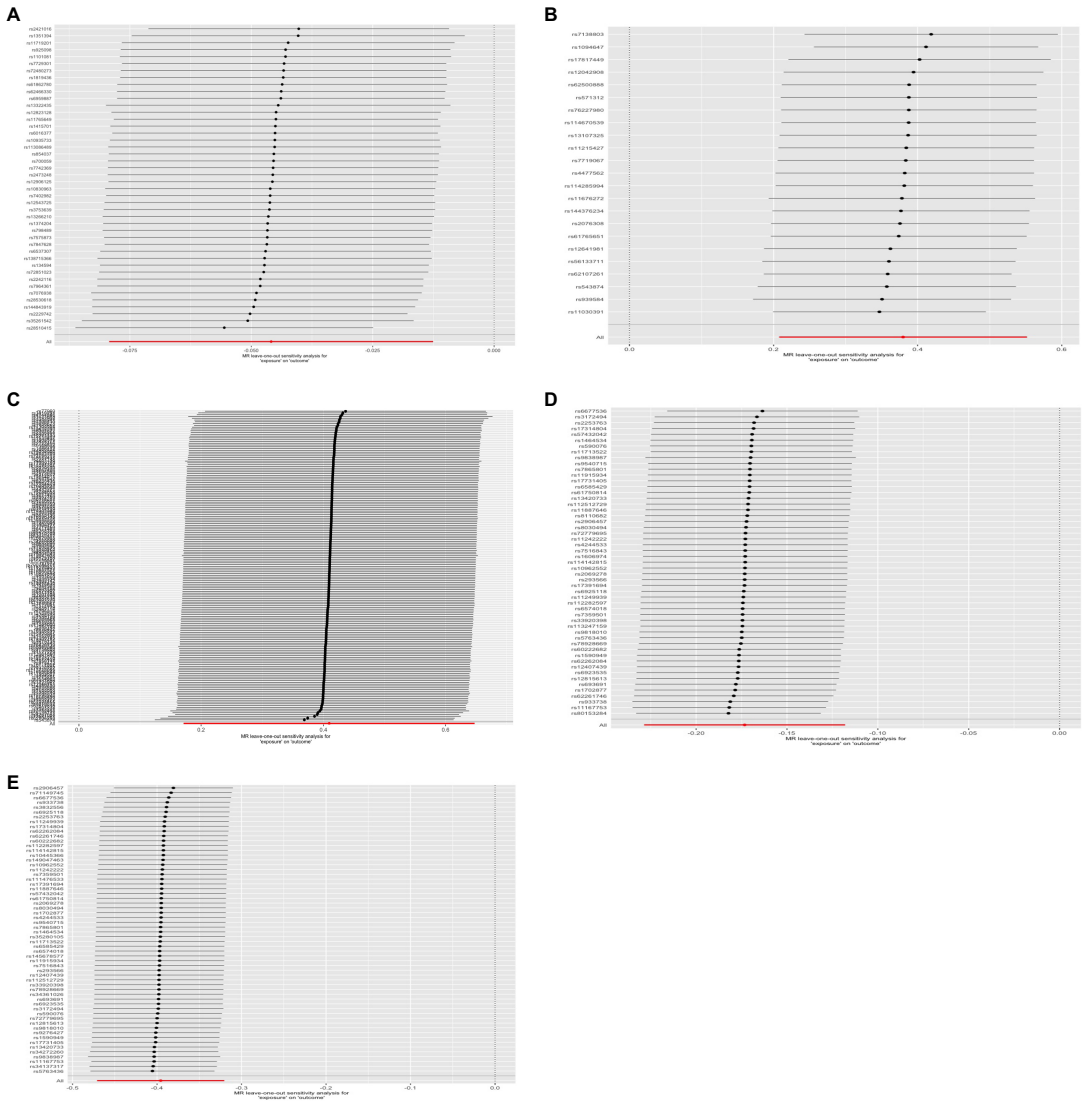
Leave-one-out analysis plot. The estimation effects are reported per SD increase in the exposure, and error bars represent 95% CI. **(A)** Lower birth weight (BW)-Alzheimer’s disease (AD); **(B)** childhood body mass index (BMI)-attention-deficit hyperactivity disorder (ADHD); **(C)** early life body size-ADHD; **(D)** age at first birth (AFB)-major depressive disorder (MDD); **(E)** AFB-ADHD; the solid dot means the causal effects of exposure on outcomes with four methods [MR-Egger (MRE); weighted median (WM); weighted mode (Wm); inverse-variance weighting (IVW); MR-Egger and Mendelian Randomization Pleiotropy RESidual Sum and Outlier (MR-PRESSO)]. The results of the binary outcomes are shown by OR [95%CI]. Numbers in bold mean *P-*values <5.00E-02, and italic and bold font means *P-*values <4.16E-03.

Four methods of TSMR (including IVW, MRE, WM, and Wm regression) were used to estimate causal associations between lower BW and three cognitive and psychiatric diseases. The results of the TSMR analysis indicated that lower BW had a positive causal effect on AD (OR_IVW_ = 1.04, 95% CI 1.01–1.08, *p* = 6.98E-03; OR_WM_ = 1.06, 95% CI 1.02–1.11, *p* = 2.69E-03; OR_MRE_ = 0.97, 95% CI 0.88–1.07, *p* = 5.18E-01; OR_Wm_ = 1.08, 95% CI 1.02–1.15, *p* = 1.43E-02). In this association, rs138715366 and rs13266210 were regarded as outlier SNPs, and the results reached the corrected threshold of *p*-value and showed statistical significance (*p* = 1.14E-03). However, there was no significant causal association between lower BW and MDD (OR_IVW_ = 1.01, 95% CI 0.98–1.13, *p* = 8.08E-01) or ADHD (OR_IVW_ = 1.12, 95% CI 0.91–1.38, *p* = 2.76E-01). There was heterogeneity in the association between lower BW and AD (Q_IVW_ = 76.781, Q_df = 43, *p* = 1.17E-03; Q_MRE_ = 71.89, Q_df = 42, *p* = 2.77E-03). No significant horizontal pleiotropy was observed in this MR, as shown by MR–Egger analysis (Egger_intercept = −2.72E-03, *p* = 9.83E-02). All results of the four methods are shown in Figure 1 and Supplementary Figure S1. The estimated effects of each exposure SNP (lower BW) on the outcomes (Supplementary Tables S9–S17) are shown both in forest and scatter plots (Figure 2 and Supplementary Figure S3; Figure 3 and Supplementary Figure S4). Directional horizontal pleiotropy was assessed by funnel plots, which are shown in Figure 4 and Supplementary Figure S5. Leave-one-out plots are shown in Figure 5 and Supplementary Figure S2.

### Two-sample Mendelian randomization analysis for the causal association between childhood BMI and three neurological and psychiatric diseases

Using the IVW method, causal relationships between childhood BMI and the risk of ADHD were confirmed. BMI in childhood was positively associated with ADHD (OR_IVW_ = 1.46, 95% CI 1.23–1.74, *p* = 1.52E-05). Similar results were observed and proven by weighted median regression methods; the findings were still sufficiently powered to exceed adjusted *p*-value thresholds after Bonferroni correction (OR_WM_ = 1.38, 95% CI 1.15–1.66, *p* = 3.61E-04; OR_MRE_ = 1.55, 95% CI 0.9–2.68, *p* = 1.29E-01; OR_Wm_ = 1.20, 95% CI 0.86–1.68, *p* = 2.96E-01).

There was evidence suggesting a causal association of children’s BMI with AD and MDD (AD: OR_WM_ = 1.03, 95% CI 1.00–1.05, *p* = 3.17E-02; OR_MRE_ = 1.10, 95% CI 1.04–1.17, *p* = 2.12E-03; MDD: OR_WM_ = 1.15, 95% CI 1.04–1.27, *p* = 7.20E-03; OR_Wm_ = 1.18, 95% CI 1.04–1.34, *p* = 1.51E-02); however, these results were not confirmed *via* IVW. Funnel plots, scatter plots, and leave-one-out plots are displayed in Figures 1–5 and Supplementary Figures S1–S5. These results suggest that directional pleiotropic effects and heterogeneity are not present in the association between childhood BMI and ADHD risk.

### Two-sample Mendelian randomization analysis for the causal association between early life body size and three neurological and psychiatric diseases

Similar to the results regarding the association between childhood BMI and ADHD, there was a strong causal effect of early life body size on ADHD (OR_IVW_ = 1.48, 95% CI 1.21–1.82, *p* = 1.29E-04; OR_WM_ = 1.42, 95% CI 1.09–1.84, *p* = 9.22E-03; OR_MRE_ = 2.35, 95% CI 1.50–3.67, *p* = 2.28E-04; OR_Wm_ = 1.51, 95% CI 1.09–2.09, *p* = 1.44E-02). After performing MR-PRESSO and deleting outliers (rs3774604, rs78444298, and rs663129) to account for the heterogeneity (*Q*_IVW_ = 538.65, *Q*_df = 267, *p* = 1.68E-20; *Q*_MRE_ = 528.62, *Q*_df = 266, *p* = 1.51E-19), the causal relationship was still observed (OR_MR-PRESSO_ = 1.47, 95% CI 1.22–1.79, *p* = 9.62E-05). There was suggestive evidence for causal relationships between early life body size and AD (OR_WM_ = 1.05, 95% CI 1.00–1.09, *p* = 2.59E-02; OR_Wm_ = 1.05, 95% CI 1.00–1.10, *p* = 4.49E-02) and MDD (OR_WM_ = 1.18, 95% CI 1.01–1.38, *p* = 3.50E-02; OR_Wm_ = 1.28, 95% CI 1.03–1.59, *p* = 1.68E-02). However, these associations did not remain significant after accounting for the heterogeneity by conducting sensitivity analyses and using the MR-PRESSO method (AD: OR = 1.02, 95% CI 0.99–1.05, *p* = 7.20E-05; MDD: OR = 1.06, 95% CI 0.95–1.18, *p* = 3.28E-01).

### Two-sample Mendelian randomization analysis for the causal association between AFB and three neurological and psychiatric diseases

Sufficient and strong evidence was obtained *via* four methods of TSMR and indicated that age at first birth had a causal effect on MDD (OR_IVW_ = 1.19, 95% CI 1.13–1.26, *p* = 8.31E-10; OR_WM_ = 1.21, 95% CI 1.14–1.28, *p* = 4.92E-10; OR_Wm_ = 1.22, 95% CI 1.09–1.37, *p* = 1.30E-03). Although unbalanced horizontal pleiotropy (MR–Egger intercept, *p* = 4.70E-06) and significant heterogeneity (Q_IVW_ = 108.87, Q_df = 52, *p* = 6.62E-06) were observed, the results remained stable after performing the MR-PRESSO procedure (OR_MR-PRESSO_ = 1.20, 95% CI 1.15–1.25, *p* = 1.96E-10) and after excluding rs2069278, rs11167753, and rs1606974. In addition, we obtained other information on the causal link between AFB and ADHD: earlier AFB was found to be associated with a higher risk of ADHD (OR_IVW_ = 1.49, 95% CI 1.38–1.60, *p* = 6.62E-25; OR_WM_ = 1.38, 95% CI 1.26–1.53, *p* = 5.79E-11; OR_Wm_ = 1.41, 95% CI 1.19–1.66, *p* = 2.13E-04), and this effect remained significant even after performing the MR-PRESSO procedure (outliers: rs7516843, rs4443016; OR_MR-PRESSO_ = 1.45, 95% CI 1.35–1.54, *p* = 1.47E-15).

### Reverse Two-sample Mendelian randomization analysis for the causal association of ADHD with childhood BMI and early life body size

To determine the reverse effects of ADHD on childhood BMI and early life body size, we conducted additional TSMR. Unfortunately, only potential and suggestive causal links were observed between ADHD and childhood BMI (OR_IVW_ = 1.08, 95% CI 1.00–1.17, *p* = 4.67E-02), and this result was not confirmed *via* other methods (OR_MRE_ = 1.26, 95% CI 0.94–1.68, *p* = 1.79E-01; OR_WM_ = 1.11, 95% CI 0.99–1.23, *p* = 5.20E-02; OR_Wm_ = 1.15, 95% CI 0.96–1.36, *p* = 1.70E-01). ADHD was also not found to have a reverse causal effect on early life body size (OR_IVW_ = 1.01, 95% CI 0.99–1.03, *p* = 7.23E-02; OR_MRE_ = 0.96, 95% CI 0.90–1.01, *p* = 1.76E-01; OR_WM_ = 1.01, 95% CI 0.99–1.03, *p* = 2.34E-01; OR_Wm_ = 1.02, 95% CI 0.99–1.06, *p* = 2.52E-01). Detailed information about these analyses is displayed in Supplementary Tables S11, S14 and Supplementary Figures S6, S7.

### Two-sample Mendelian randomization analysis for the causal associations of childhood BMI, early life body size, and AFB with sex-specific ADHD

A sex-specific TSMR was performed to examine the causal associations of childhood BMI, early life body size, and AFB with ADHD among male and female participants separately. We observed the causal relationship between early life body size and ADHD among male participants (OR_MR-PRESSO_ = 1.50, 95% CI 1.17–1.90, *p* = 1.28E-03) but not among female participants. For childhood BMI and AFB, the results were consistent across the sexes. Detailed information about these analyses is displayed in Supplementary Tables S12, S15, S17 and Supplementary Figures S8–S12.

### Multivariable Mendelian randomization

Performing multivariable mendelian randomization (MVMR) resulted in the elimination of influence estimates in the previous LBW and AD MR analysis after adjusting for adult BMI. In the MVMR analysis controlling for BMI, there was no evidence of a causal link between LBW and AD (OR_MVMR_ = 0.97, 95% CI 0.92–1.02, *p* = 3.00E-01) (Supplementary Table S18).

## Discussion

Based on the theory of Barker, we selected four variables assessed between birth (AFB and BW) and the age of 10 years (early life body size and childhood BMI) from aggregated GWAS data, and we examined their causal effects on ADHD in adolescence, MDD in adulthood, and AD in old age. Our findings strongly suggest that three early indicators—i.e., higher childhood BMI, larger early life body size, and earlier AFB—predict a higher risk of ADHD. We also observed causal relationships between lower BW and a higher incidence of AD, earlier AFB, and a higher occurrence of MDD. To the best of our knowledge, this is the first systematic study based on genetic information to study the possible causal relationships between early life course-related characteristics and neurological and psychiatric diseases across the whole life span.

Challenges in early life, including perinatal and early postpartum periods, may have a profound impact on the neural development of offspring (56). The perinatal period is the most sensitive period to nutritional status in the life process. Interruptions to the concentrated flow of fetal nutrition during this time, such as premature delivery, malnutrition, or placental insufficiency, may lead to LBW. Some studies have shown an association between LBW and a high risk of adult mental illness (57, 58). Observational evidence has shown that smaller birth size and LBW are significantly associated with cognitive and motor impairment, which have lower composite cognitive scores (*β* = −0.12, 95% CI[−0.19, −0.05], *p* = 0.001) in later life compared to those with normal BW (59–62). Other results also indicate that LBW newborns exhibit developmental alterations in gene expression involved in cell differentiation, neurogenesis, and neurodegeneration (63). Although our results provide statistically significant results demonstrating the causal link between LBW newborns and AD (OR_IVW_ = 1.04, 95% CI 1.01–1.08, *p* = 6.98E-03), there is still a lack of clarity regarding the etiologies and pathogeneses of AD. After adjusting for genetic prediction of LBW and BMI initiation by using MVMR, there was no direct causal association between LBW and AD (OR_MVMR_ = 0.97, 95% CI 0.92–1.02, *p* = 3.00E-01). Large meta-analysis studies also did not state that lower BW was an absolute risk factor for AD, but high BMI in late life was a level A strong risk factor for AD (64, 65). We did not find any correlation between LBW and MDD or ADHD (MDD: OR_IVW_ = 1.01, 95% CI 0.98–1.13, *p* = 8.08E-01; ADHD: OR_IVW_ = 1.12, 95% CI 0.91–1.38, *p* = 2.76E-01). Some previous epidemiological studies found a higher risk of depression among preterm birth and LBW individuals than among controls (OR = 2.86, 95% CI [1.73–4.73]; OR = 1.39, 95% CI 1.21–1.60) (9, 66). Furthermore, LBW individuals reported more severe symptoms of ADHD (*r* = –0.15) (67) as well as an increased risk of ADHD (68, 69).

Another prominent and serious issue is obesity, especially in adolescents. As of 2019, the World Obesity Federation (WOF) estimated that there would be 206 million obese children and adolescents (ranging from 5 to 19 years) in 2025, and this number is expected to increase to 254 million in 2030 (70). The cooccurrence of ADHD and obesity is caused by both genetic and prenatal environmental origins (71). A series of studies have revealed a significant association between overweight/obesity and children/adolescents ADHD (overweight: 18.8–31.2%; obesity: 13.5–19.3%) (72, 73) but a weak relationship in adults with obesity (74, 75). To explain this correlation, Albayrak (76) found that there is a common background of genetics (MAP2K5, GPRC5B, and CADM2) between ADHD and obesity, while Cortese S. (77) revealed that inflammatory cytokines may play a connective role between them. One study announced that there was no reliable and robust association between ADHD and BMI at any age or time point (75). Herein, the strong correlation between childhood obesity and ADHD was due to the use of two easy and common measurements: childhood BMI and early life body size (OR_IVW_ = 1.46, 95% CI 1.29–1.66, *p* = 9.13E-06; OR_IVW_ = 1.47, 95% CI 1.22–1.79, *p* = 9.62E-05). Furthermore, considering that the association was complex and bidirectional rather than simple and unidirectional (74, 78), we performed reverse TSMR, and there were no positive findings. In addition, sex/gender differences in ADHD have been reported (79), but the correlation between childhood obesity and ADHD remains unclear. Nigg JT (75) and Fliers EA (80) found that ADHD was associated with obesity in adolescent girls but not in children or boys, while Jongpitakrat K (78) and Aguirre Castaneda RL (81) stated that male children and adolescents with obesity were significantly associated with an increased prevalence of ADHD. Thus, we also provided evidence that a larger early life body size could increase the risk of ADHD in male participants (OR _MR-PRESSO_ = 1.50, 95% CI 1.17–1.90, *p* = 1.28E-03) but not in female participants. Many researchers have focused on the association between adult and even middle-life obesity and AD (82–85), which could alter cognitive and brain reserve (86), but very few studies have examined this issue in children. One study reported that there was no evidence from individual SNP or polygenic scores, indicating a relationship between BMI and increased AD risk (87), consistent with our results for childhood BMI (OR_IVW_ = 1.02, 95% CI 0.99–1.04, *p* = 1.54E-01) and early life body size (OR_MR-PRESSO_ = 1.02, 95% CI 0.99–1.05, *p* = 7.20E-02). For MDD, studies supported that obesity increased the risk of the onset of depression (OR = 1.55; 95% CI 1.22–1.98; *p* < 0.001) (88–90), and obese children and adolescents were more likely to have MDD (OR = 1.851, 95% CI 1.41–2.43) (91). However, Jokela (92) considered an adverse metabolic profile rather than using obesity as a risk factor for depression. We did not obtain any evidence for childhood BMI or early life body size.

Preterm birth adults exhibited a 1.14- to 1.3-fold risk of being diagnosed with a depressive disorder (93–96), especially extreme preterm birth at <28 weeks (97), and our results are consistent with these observational studies (OR_MR-PRESSO_ = 1.20, 95% CI 1.15–1.25, *p* = 1.96E-10). Many researchers have reported that preterm-born children have an increased incidence of ADHD (RR: ranged from 1.26 to 2.64) (69, 98–101), as well as our results (OR_MR-PRESSO_ = 1.45, 95% CI 1.35–1.54, *p* = 1.47E-15). No causal link was found between AFB and AD, although some results reported that preterm birth could reduce cognitive scores (98).

The greatest advantage of this study lies in the use of TMSR, a new analytical method. Correlation analysis can provide us with some research ideas, but its evidence is limited. Sometimes, due to the existence of confounding factors, false correlations can occur. Four early life course-related characteristics [i.e., birth weight (BW), child body mass index (BMI), early body shape, and age at birth (AFB)] were used as exposure IVs to analyze their causal relationships with neurological and psychiatric diseases. SNP or whole-genome sequencing can be used as a tool to avoid the influence of the external environment and improve the strength of causal inferences. Of course, this study focuses on the adverse effects of childhood obesity and premature delivery on ADHD in the future, but the specific mechanism has yet to be fully elucidated. An association between exposure and disease is epigenetics. For example, the environment triggers epigenetic changes to produce unique adult phenotypes, which is also a recent hot research topic in this field.

Several limitations inevitably existed in our study. First, most of the GWAS data were obtained from European participants, thus limiting the ability to generalize these results to other races, sexes, or areas. Second, the selected exposure IVs were not divided based on sex, which may lead to collider bias with respect to ADHD (102).

## Conclusion

The results of our study indicate that childhood adiposity and preterm delivery have a causal effect on ADHD in adolescence; that LBW may not be associated with the risk of AD after adjusting for adult BMI; and that preterm birth is associated with a higher incidence of MDD symptoms in adulthood.

## Data availability statement

The original contributions presented in the study are included in the article/Supplementary material, further inquiries can be directed to the corresponding authors.

## Ethics statement

Ethical review and approval was not required for the study on human participants in accordance with the local legislation and institutional requirements. Written informed consent to participate in this study was provided by the participants’ legal guardian/next of kin.

## Author contributions

RH and JM envisaged and designed the study. RH, JM, and KZ obtained and analyzed the datasets. RH finalized the main manuscript, whereas KZ, QL, XL, and JS received funding and revised the manuscript. All authors reviewed and approved the final version of the manuscript.

## Funding

The National Natural Science Foundation of China (82088102), the Collaborative Innovation Program of Shanghai Municipal Health Commission (2020CXJQ01), the Shanghai Frontiers Science Center of Reproduction and Development, the CAMS Innovation Fund for Medical Sciences (2019-I2M-5-064), the National Key Research and Development Program of China (2022YFC2703000), the National Nature Science Foundation of China (82171613), and a project supported by the Scientific Research Fund of Zhejiang Provincial Education Department (Y202250855) provided financial support.

## Conflict of interest

The authors declare that the research was conducted in the absence of any commercial or financial relationships that could be construed as a potential conflict of interest.

## Publisher’s note

All claims expressed in this article are solely those of the authors and do not necessarily represent those of their affiliated organizations, or those of the publisher, the editors and the reviewers. Any product that may be evaluated in this article, or claim that may be made by its manufacturer, is not guaranteed or endorsed by the publisher.
