# Supplementary material for: The early life course-related traits with three psychiatric disorders: A two-sample Mendelian randomization study
Source: Front Psychiatry. 2023 Mar 21;14:1098664. doi: 10.3389/fpsyt.2023.1098664 (PMC10070876; doi:10.3389/fpsyt.2023.1098664)
Supplement: Supplementary file 1 [file Image_1.pdf]

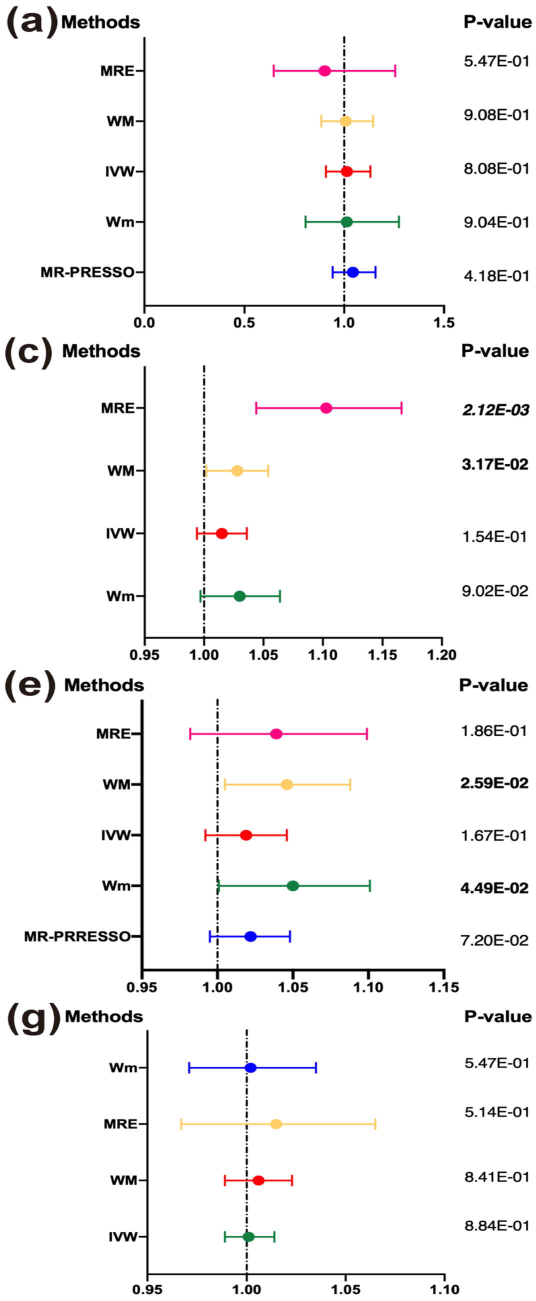

1 **Supplementary Figure 1. The results of four different methods of MR analysis.** (The MR analysis showing the effect of the exposure SNPs on the outcomes. **a–g**: [**a**] lower birth weight (BW)- major depressive disorder (MDD); [**b**]BW- attention deficit hyperactivity disorder (ADHD); [**c**] childhood body mass index (BMI)-AD;[**d**] childhood BMI-MDD;[**e**] early life body size-Alzheimer' disease (AD);[**f**] early life body size-MDD;[**g**] AFB-AD;The solid dot means the causal effects of exposure on outcomes with four methods (MR-Egger (MRE); weighted median (WM); weighted mode (Wm); inverse variance weighting (IVW); MR-Egger and Mendelian Randomization Pleiotropy RESidual Sum and Outlier (MR-PRESSO). The results of the binary outcomes are shown by OR [95%CI]. Numbers in bold mean *p*-values < 5.00E-02, and italic and bold font means *p*-values < 4.16E-03**)**

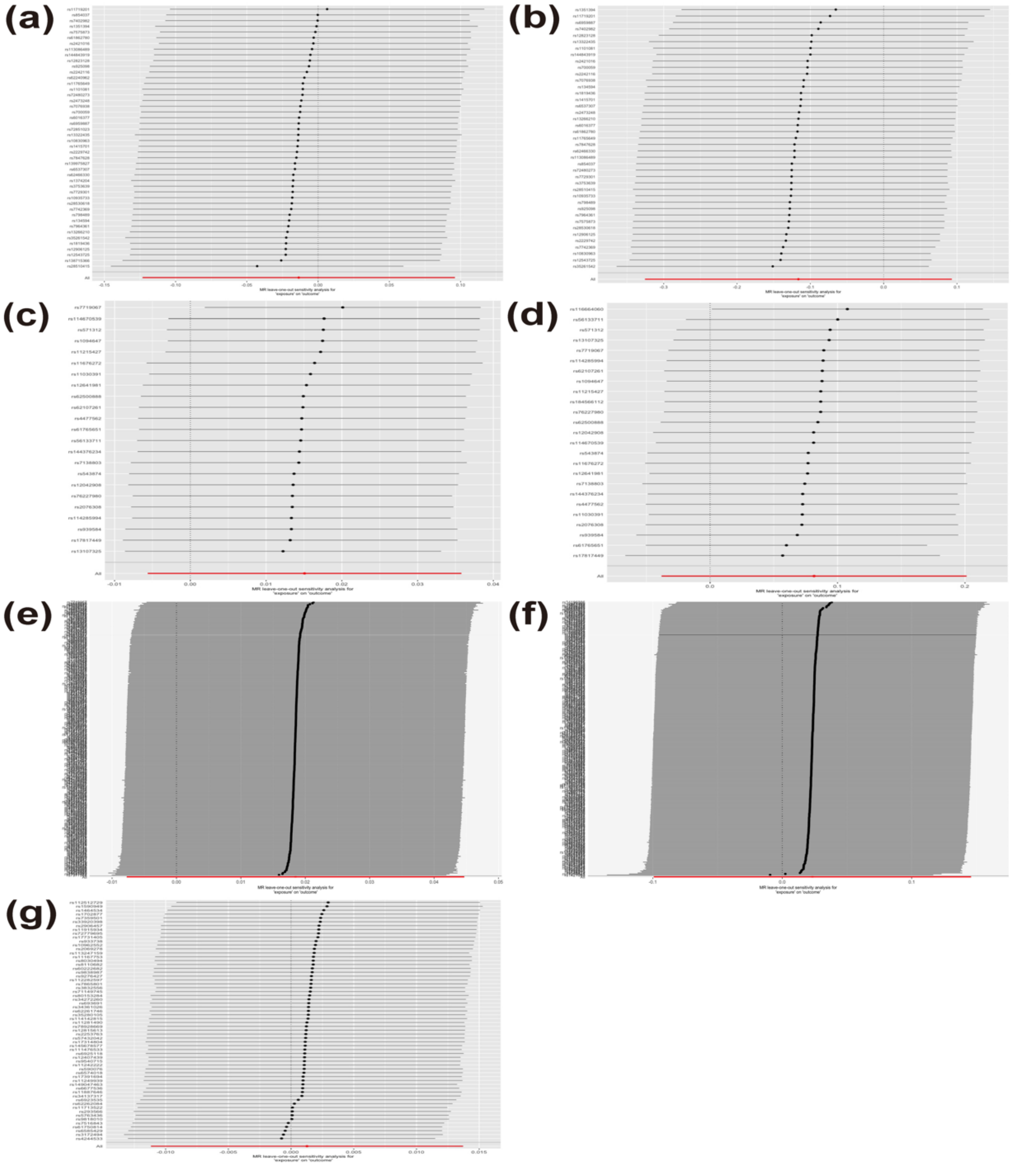

Supplementary Figure 2. The leave-one-out analysis plot. (The estimation effects are reported per SD increase in the exposure, and error bars represent 95% CI**)**

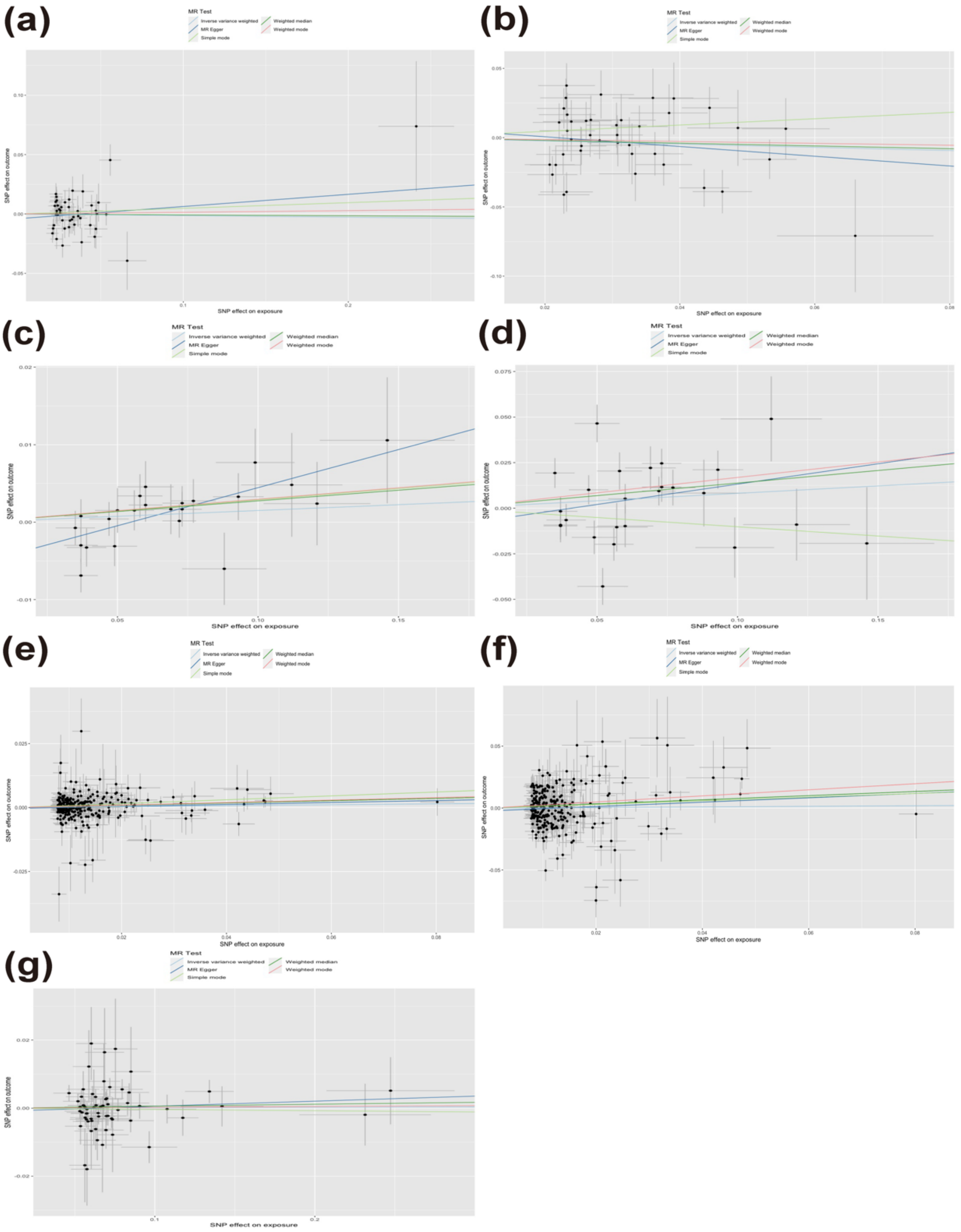

3 **Supplementary Figure 3. The results of the scatterplot**

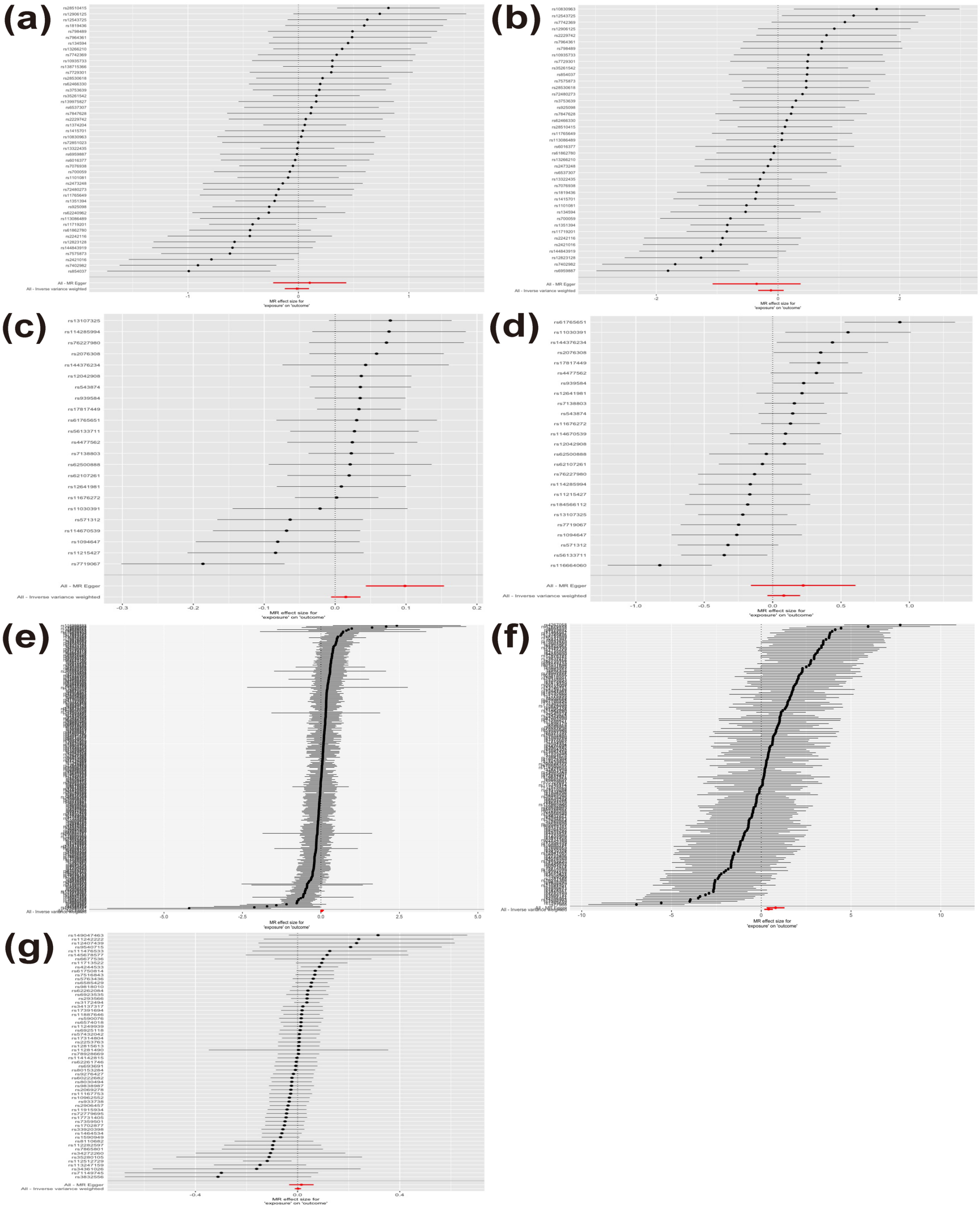

4 **Supplementary Figure 4. The results of the forest plot**

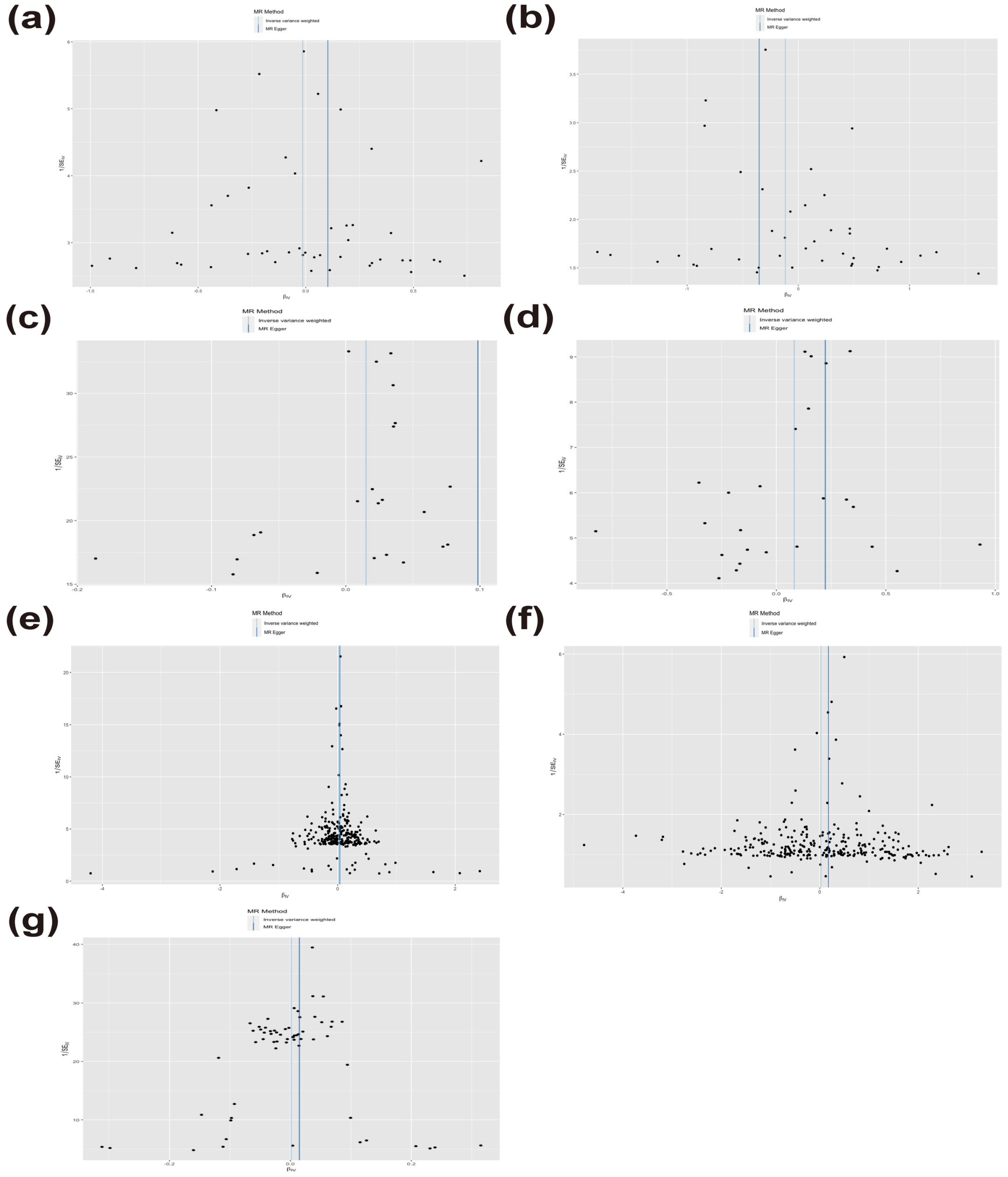

5 **Supplementary Figure 5. The results of the funnel plot**

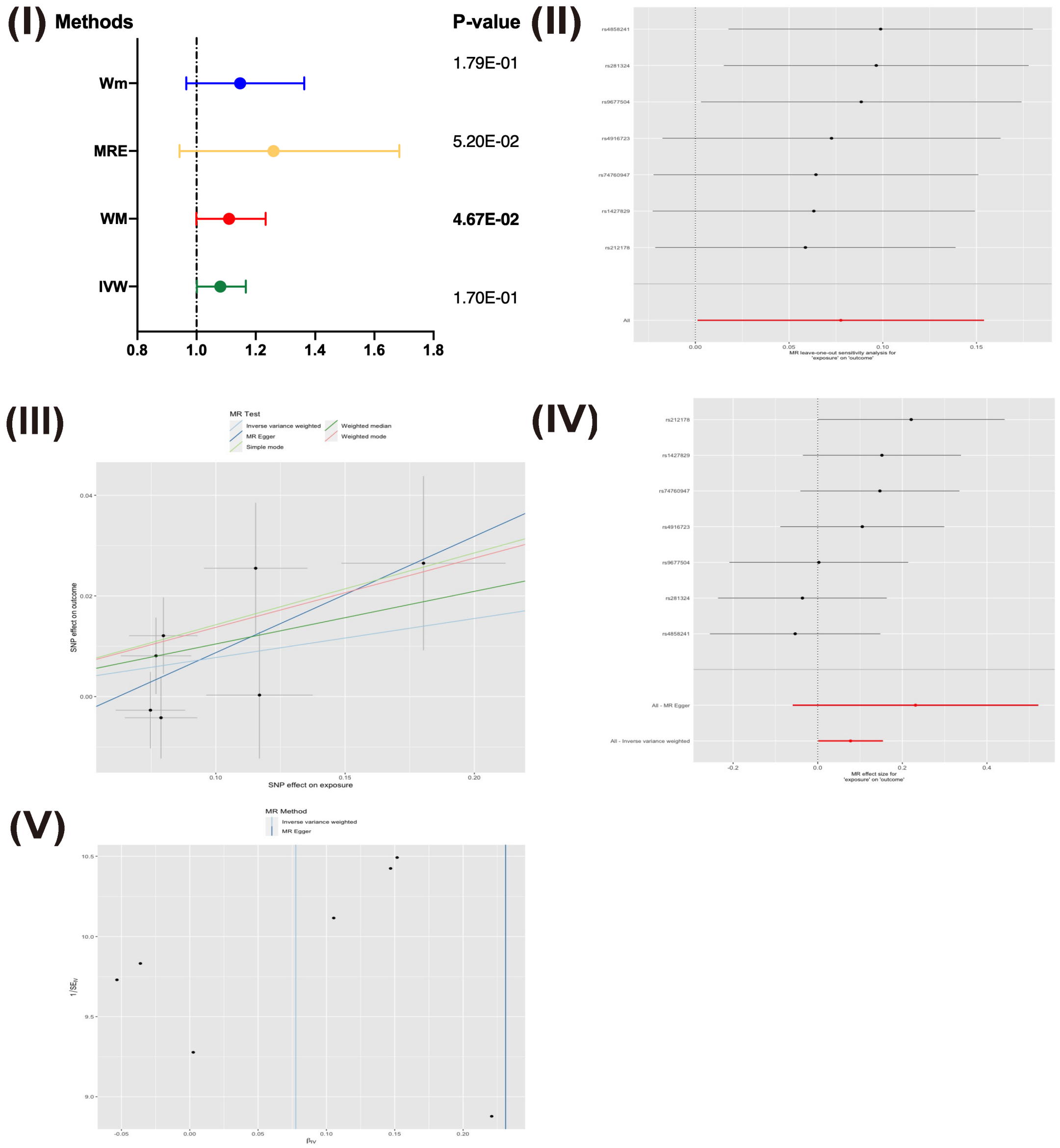

6 **Supplementary Figure 6. The results of reverse MR analysis between ADHD and childhood BMI.** (The MR analysis showing the effect of the exposure SNPs on the outcomes. **I-V**: [**I**] MR results of four different methods; The solid dot means the causal effects of exposure on outcomes with four methods (MR-Egger (MRE); weighted median (WM); weighted mode (Wm); inverse variance weighting (IVW); MR-Egger and Mendelian Randomization Pleiotropy RESidual Sum and Outlier (MR-PRESSO); [**II**] The leave-one-out analysis plot. (The estimation effects are reported per SD increase in the exposure, and error bars represent 95% CI); [**III**] The results of the scatterplot; [**IV**] The results of the forest plot; [**V**] The results of the funnel plot; Numbers in bold mean p-values < 5.00E-02, and italic and bold font means p-values < 4.16E-03);

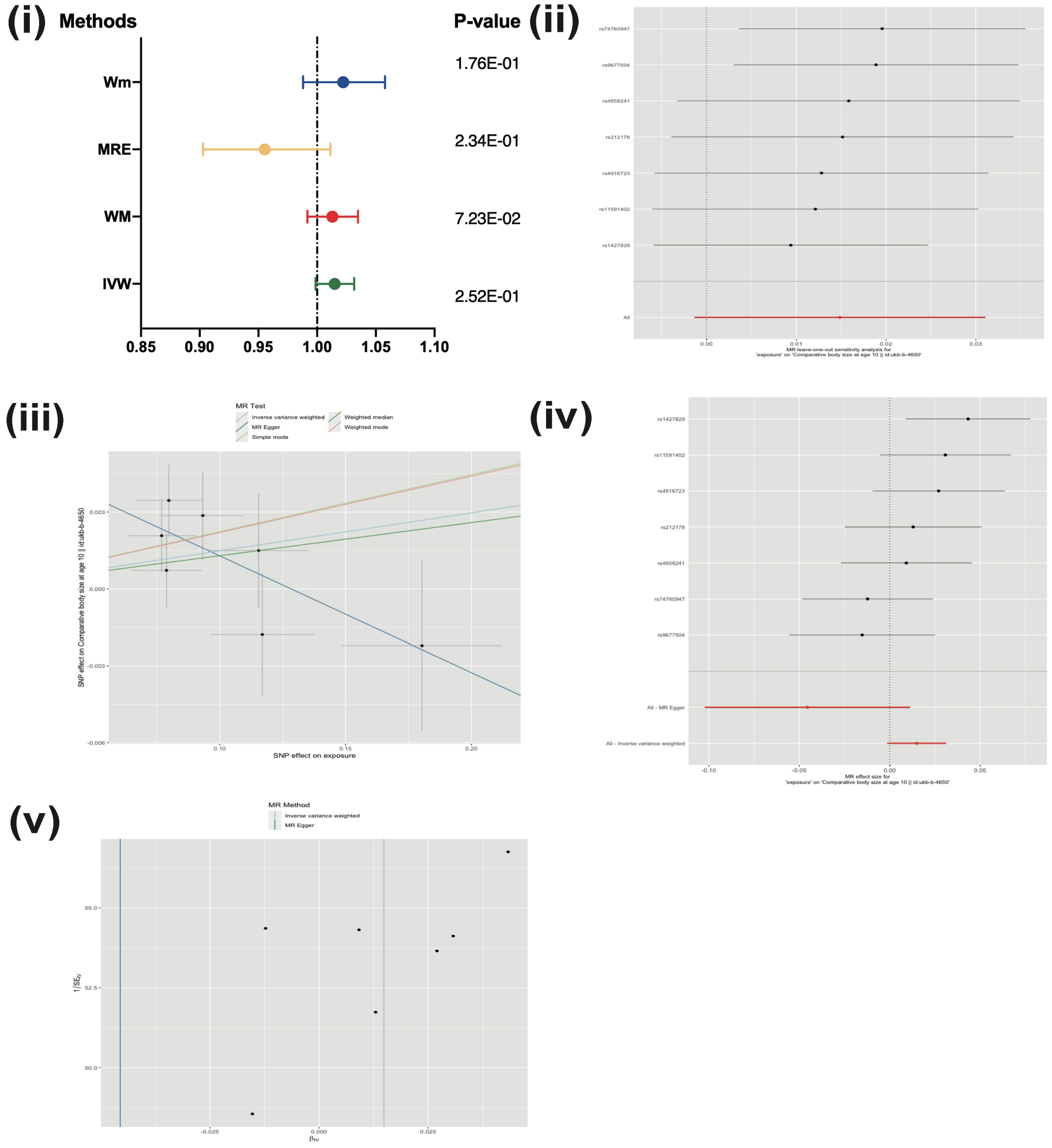

Supplementary Figure 7. The results of reverse MR analysis between ADHD and early life body size. (The MR analysis showing the effect of the exposure SNPs on the outcomes. **i-v**: [**i**] MR results of four different methods; The solid dot means the causal effects of exposure on outcomes with four methods (MR-Egger (MRE); weighted median (WM); weighted mode (Wm); inverse variance weighting (IVW); MR-Egger and Mendelian Randomization Pleiotropy RESidual Sum and Outlier (MR-PRESSO); [**ii**] The leave-one-out analysis plot. (The estimation effects are reported per SD increase in the exposure, and error bars represent 95% CI); [**iii**] The results of the scatterplot; [**iv**] The results of the forest plot; [**v**] The results of the funnel plot; Numbers in bold mean p-values < 5.00E-02, and italic and bold font means p-values < 4.16E-03)

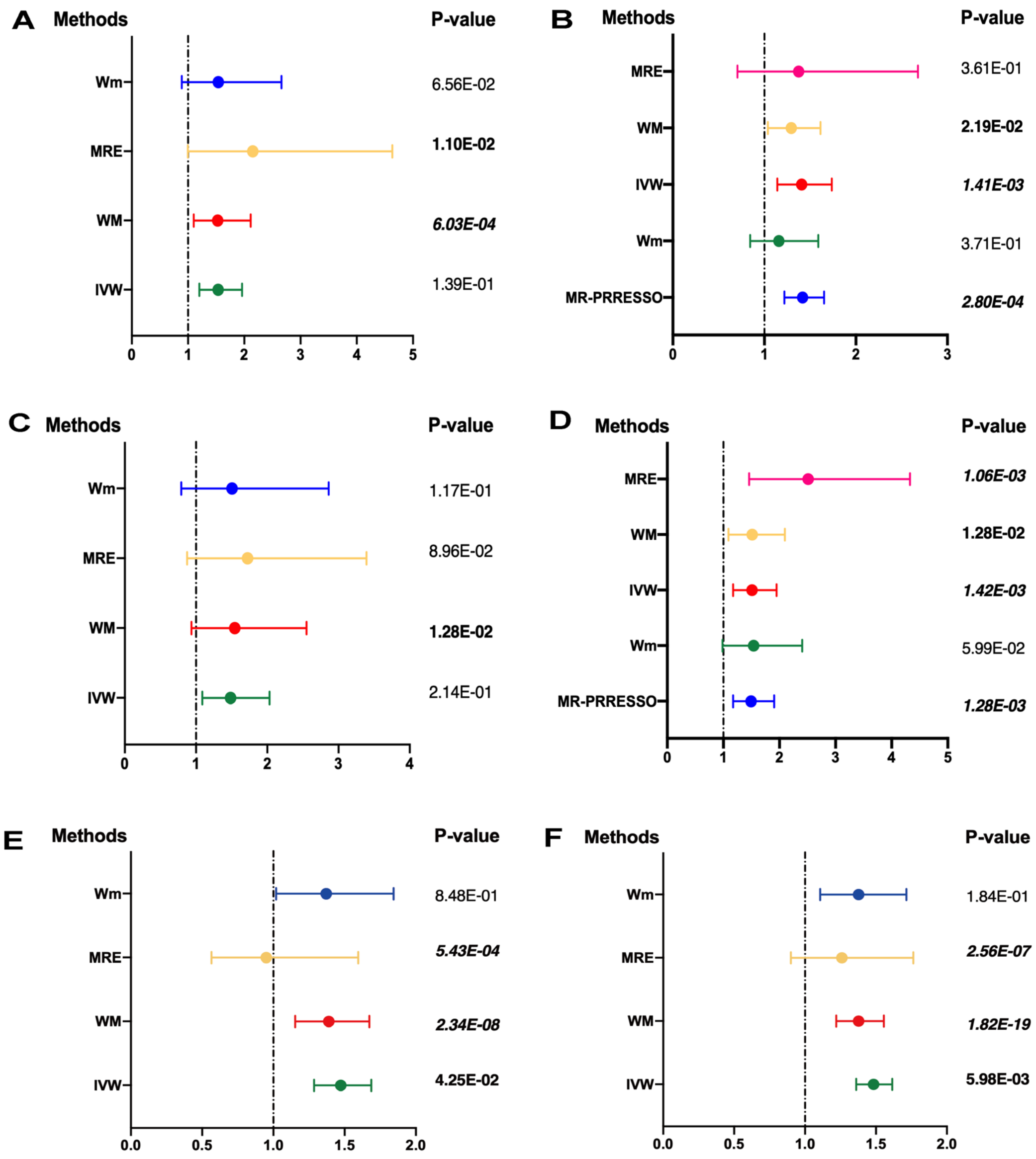

8 **Supplementary Figure 8. The results of four different methods of MR analysis.** (The MR analysis showing the effect of the exposure SNPs on the outcomes. A-**F**: [**A**] childhood body mass index (BMI)- attention deficit hyperactivity disorder (ADHD)-female only; [**B**] childhood body mass index (BMI)-attention deficit hyperactivity disorder (ADHD)-male only; [**C**] early life body size- attention deficit hyperactivity disorder (ADHD)-female only;[**D**] early life body size- attention deficit hyperactivity disorder (ADHD)-male only;[**E**] earlier age at first birth(AFB) - attention deficit hyperactivity disorder (ADHD)-female only;[**F**] earlier age at first birth(AFB) - attention deficit hyperactivity disorder (ADHD)-male only;

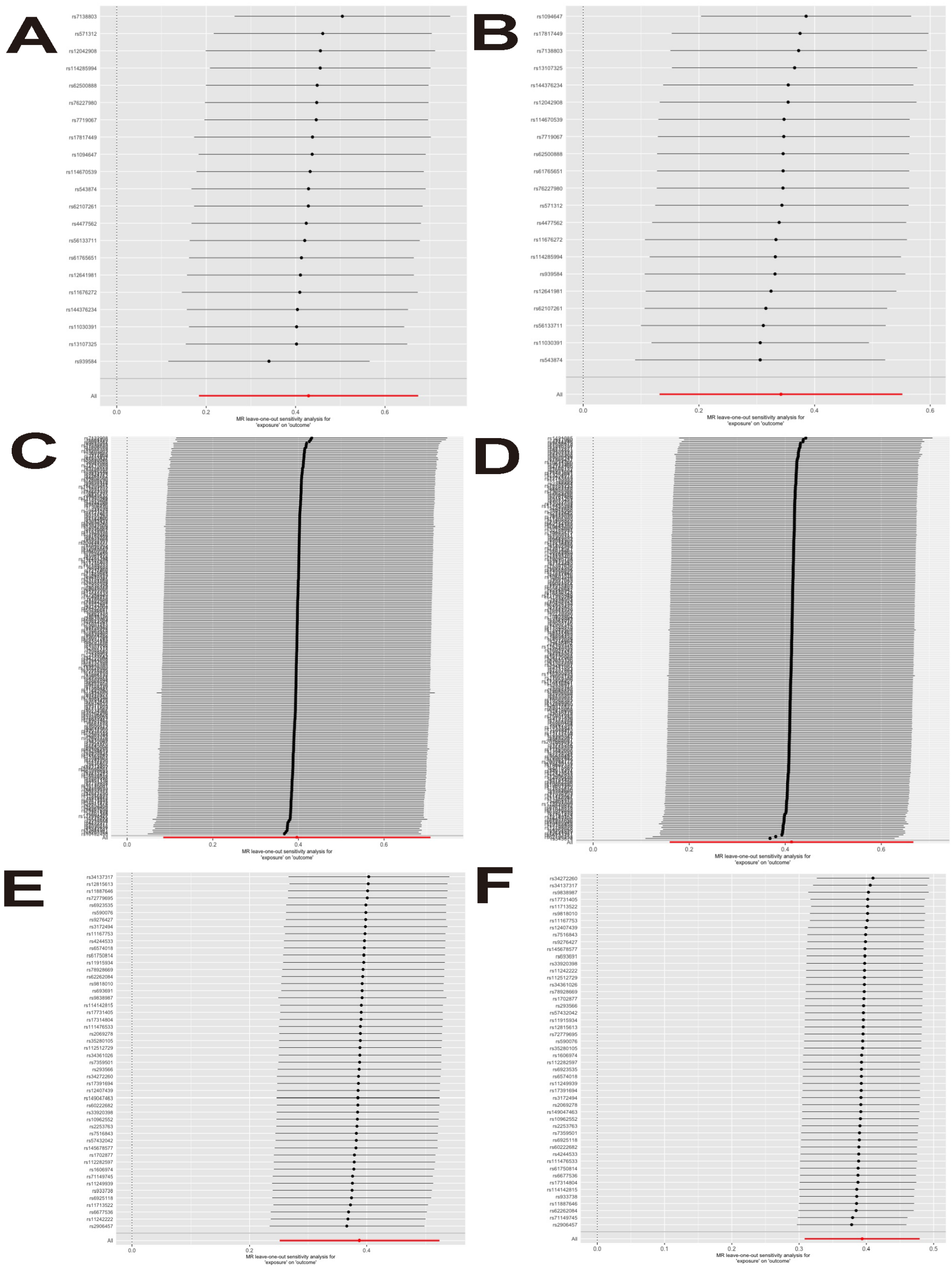

9 **Supplementary Figure 9. The leave-one-out analysis plot. (**The estimation effects are reported per SD increase in the exposure, and error bars represent 95% CI**)**

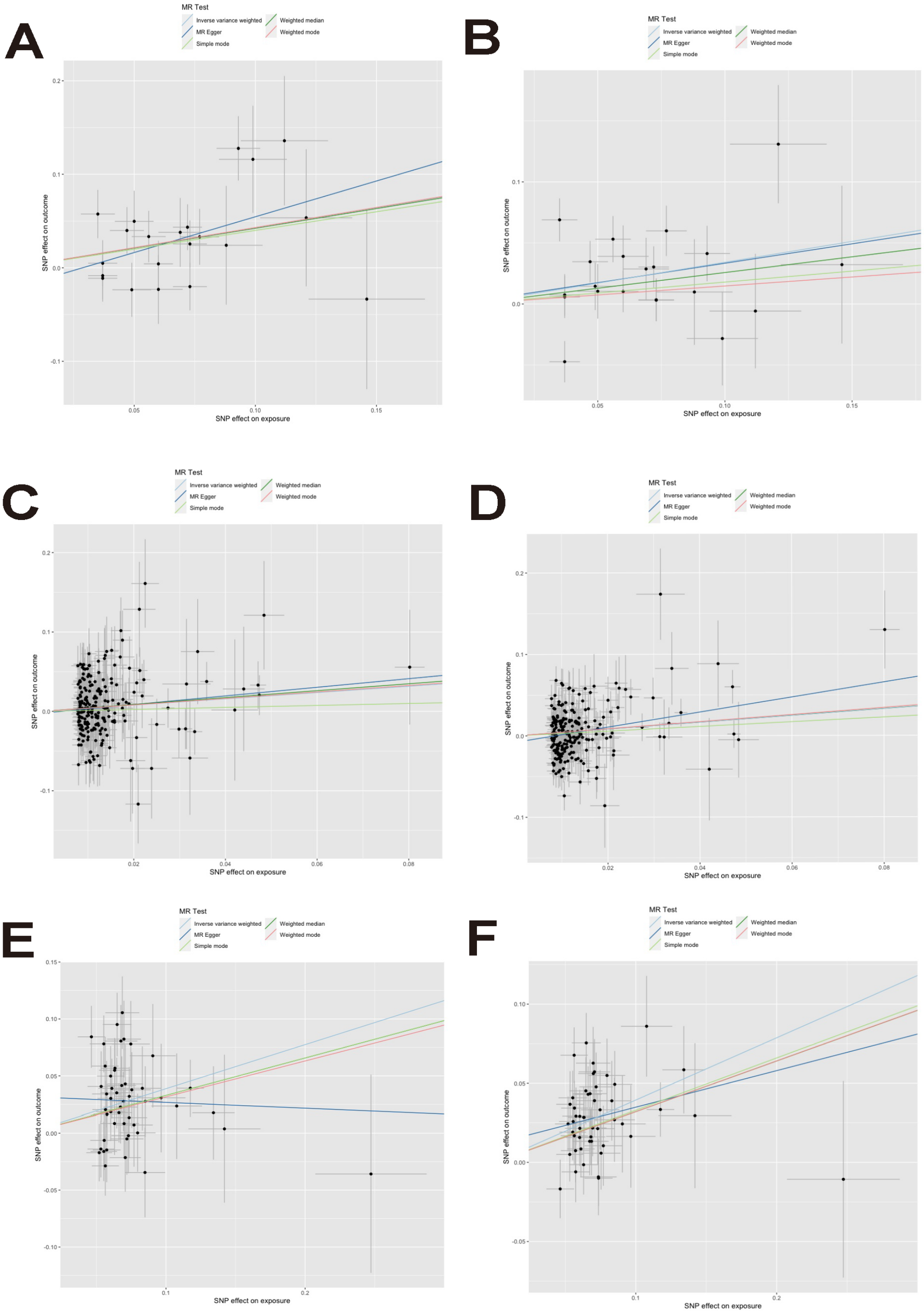

10 **Supplementary Figure 10. The results of the scatterplot**

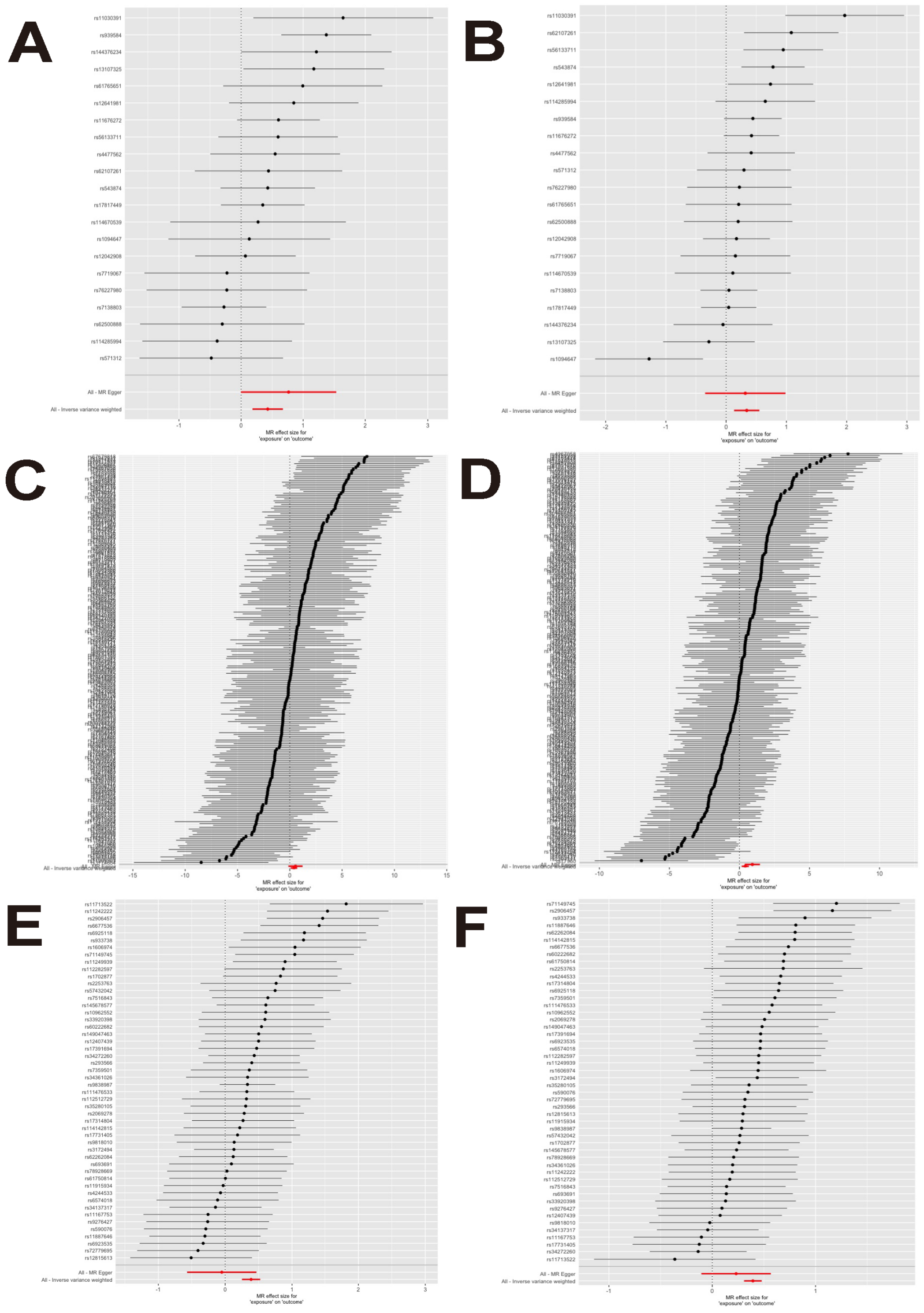

11 **Supplementary Figure 11. The results of the forest plot**

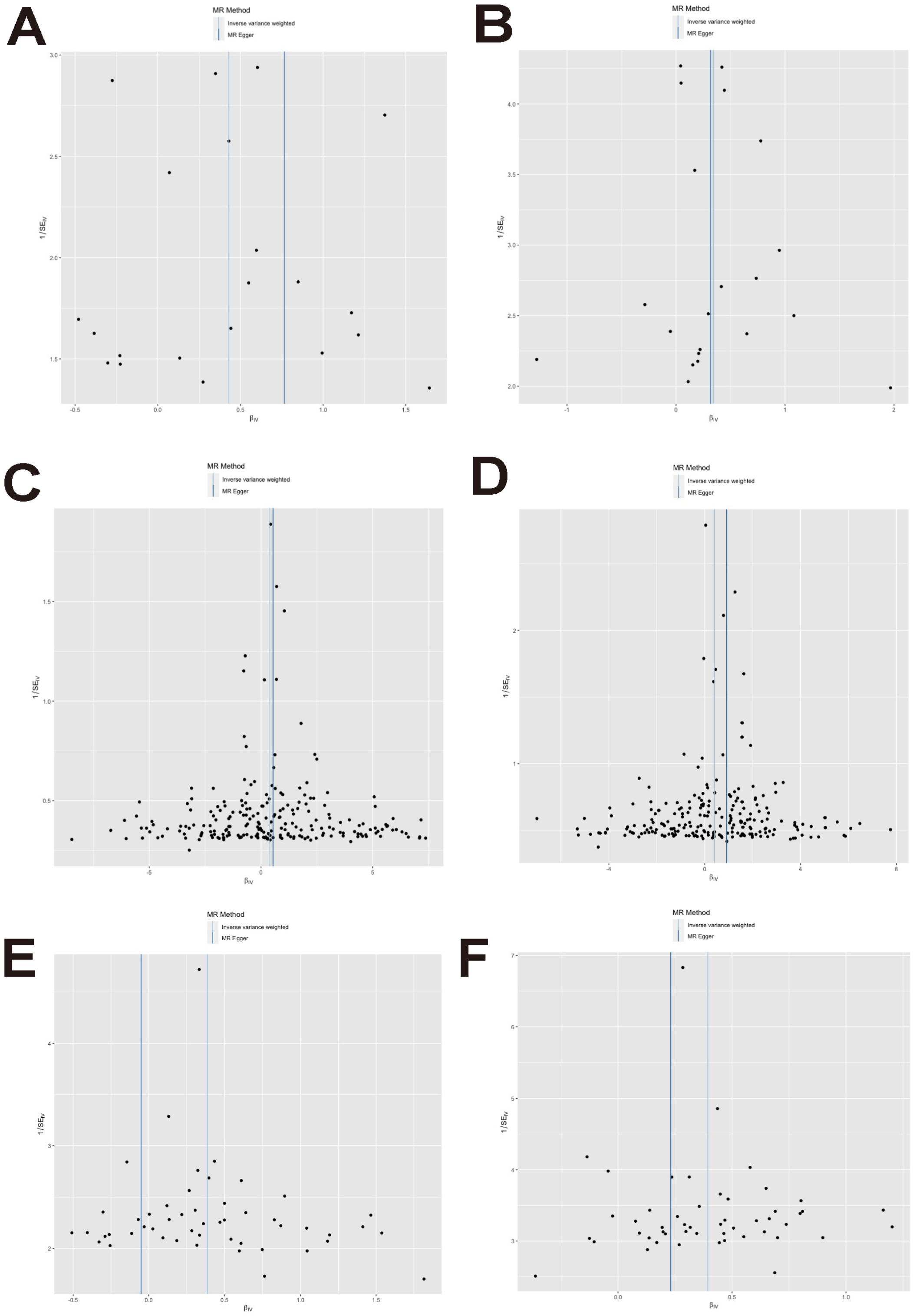

12 **Supplementary Figure 12. The results of the funnel plot**
